# Use of Headphones and Its Impact on Hearing: A Cross-Sectional Survey of Medical, Nursing, and Pharmacy Students

**DOI:** 10.7759/cureus.96900

**Published:** 2025-11-15

**Authors:** Gursimrat Kaur, Aditya Anand, Priyanko Chakraborty, Tamanna Pravin, Mohammed Khalilullah, Rishi Tarafdar, Devanshu Raj, Subhasish Mukherjee

**Affiliations:** 1 Otolaryngology - Head and Neck Surgery, Mata Gujri Memorial Medical College and LSK Hospital, Kishanganj, IND

**Keywords:** headphone use, hearing health, heathcare sector, medical students, noise exposure, noise induced hearing loss, nursing students, pharmacy students

## Abstract

Background

Personal audio device use among healthcare students is widespread, raising concerns about noise‑induced hearing loss.

Objective

To evaluate patterns of headphone use, quantify noise exposure, and examine their association with auditory symptoms and sensorineural hearing loss (SNHL) among medical students pursuing the Bachelor of Medicine, Bachelor of Surgery (MBBS) degree, pharmacy, and nursing students.

Methodology

A cross‑sectional study surveyed 250 undergraduate medical, pharmacy, and nursing students (aged 18-30 years, mean 20.8 ± 2.1) at MGM Medical College and Hospital, Kishanganj. Participants completed a questionnaire on headphone type, listening duration, and volume, followed by otoscopic examination and pure‑tone audiometry. Associations between device‑use patterns and auditory outcomes were tested using the chi‑square test.

Results

Regular headphone use was reported by 210 students (84.0%). Earbuds were the most common device (91 users, 43.3%), followed by wired earphones (58, 27.6%), Bluetooth earphones (47, 22.4%), and headsets (14, 6.7%); choice differed significantly by program (χ² = 40.88, *P* < 0.001). Daily listening lasted <2 hours for 92 users (43.8%), 2-4 hours for 85 (40.5%), 4-6 hours for 25 (11.9%), and >6 hours for 8 (3.8%). Volume was kept below 50% of maximum by 100 users (47.6%), at 50%-80% by 103 (49.0%), and above 80% by 7 (3.4%). Headache was the most common complaint (62 students, 24.8%), followed by eye watering (53, 21.2%), intolerance to loud sounds (37, 14.8%), and ear pain (36, 14.4%). Symptoms were more frequent with both longer duration and higher volume: all students who listened at >80% volume for >4 hours experienced multiple symptoms, whereas only about one‑fifth of those listening for the same duration at <50% volume reported any. SNHL was detected in nine students (3.6%, 95% confidence interval (CI) 1.9%-6.7%), seven with mild and two with moderate impairment; eight of these nine cases displayed the 4-6 kHz notch characteristic of noise‑induced damage.

Conclusions

Unsafe listening practices are common among healthcare students. Higher listening volumes and extended daily use were associated with increased auditory symptoms and early SNHL. Volume regulation appears more critical than duration alone in reducing risk. Educational initiatives and routine screening are warranted, particularly for MBBS students, to protect hearing health.

## Introduction

In recent years, the use of personal audio devices such as headphones and earphones has become increasingly prevalent among students, particularly in demanding academic fields related to health care. These devices are used not only for entertainment but also for educational purposes, including listening to lectures, participating in discussions, and attending virtual learning sessions. While convenient, this growing dependence on headphones raises serious concerns regarding noise exposure and its potential impact on hearing health [1].

Many commercially available headphones and earphones have the potential to generate sound levels exceeding 125 decibels (dB) [2], far surpassing the recommended safe listening threshold [3]. Extended use of these devices at elevated volume levels can lead to cumulative trauma to the delicate cochlear hair cells, ultimately resulting in sensorineural hearing loss (SNHL) - particularly affecting the perception of high-frequency sounds. Such damage is often permanent and may go unnoticed in early stages, making it a silent but serious health concern [4].

Globally, the prevalence of disabling hearing loss among adults is estimated to be around 16%, with regional variations ranging from 7% to as high as 21%, depending on demographic and environmental factors [5,6]. Despite these concerning statistics, hearing loss-especially when caused by recreational noise exposure-remains underrecognized and underreported, particularly in younger populations.

The global shift in education delivery methods, especially after the COVID-19 pandemic, significantly altered students' learning environments [7]. With the sudden move from traditional classroom-based instruction to online platforms, students found themselves engaging in prolonged screen time and relying heavily on headphones for effective communication and concentration. This change, though essential for academic continuity, may have inadvertently contributed to increased noise exposure and heightened the risk of hearing impairment.

College students may not be fully aware of the long-term consequences of unsafe listening practices. Continuous exposure to high-volume sound through headphones, combined with a lack of knowledge about safe usage guidelines, makes this population particularly vulnerable to auditory damage.

This study aims to explore the patterns of headphone use, assess the extent of noise exposure, and evaluate the level of hearing-related risks and problems among medical, pharmacy, and nursing students. With the increasing use of personal audio devices in academic and recreational settings, the study investigates how listening habits may be contributing to early signs of hearing impairment. By addressing this often-overlooked aspect of student well-being, the research seeks to provide meaningful insights and promote preventive strategies for hearing conservation.

## Materials and methods

A cross-sectional study was conducted to evaluate headphone use and noise exposure among undergraduate students enrolled in the Bachelor of Medicine, Bachelor of Surgery (MBBS), Pharmacy, and Nursing programs. The study spanned six months in the Outpatient Department (OPD) of MGM Medical College and Hospital, Kishanganj. Eligible participants were students aged 18-30 years who provided written informed consent. Exclusion criteria included pre-existing hearing loss, hearing aid use, prior ear surgery, or inability to complete the study process.

Sample size calculation

Because the target population was students, the required sample size was calculated using a pooled prevalence of hearing loss among college students (19%) reported by a recent meta-analysis [8]. The single-proportion formula was applied: *n* = *Z*² × *p*(1 − *p*)/*d*², with *Z* = 1.96 (95% confidence), *p* = 0.19, and *d* = 0.05. This yielded *n* = 236.5, rounded to 236. Allowing for non-response, 250 students were enrolled.

Data collection

The structured questionnaire was developed by the authors after a literature review and pilot-tested among 20 students for internal consistency before full-scale administration. It captured demographics, headphone usage (duration, frequency, type, and volume), and noise exposure history (Appendix). Clinical otoscopic examination was performed to exclude external or middle ear pathology.

Audiometric assessment

All participants then underwent pure tone audiometry (PTA) in a sound-treated room using a calibrated ALPS AD2100 clinical audiometer (ALPS, New Delhi, India; ANSI S3.6-2018 standard), with TDH-39 supra-aural headphones (Telephonics Corporation, Farmingdale, NY). Tests were performed by trained audiologists approximately 24 hours after the last headphone use to minimize temporary threshold shifts. Hearing thresholds were assessed from 250 to 8,000 Hz in both ears, and results were interpreted according to standard criteria to identify hearing loss.

Statistical analysis

Data were recorded in a secure database and analyzed using IBM SPSS Statistics for Windows, Version 26.0 (IBM Corp., Armonk, NY). Descriptive statistics were used for baseline characteristics, and inferential tests (chi-square test for categorical variables, and independent t-test or analysis of variance (ANOVA) for continuous variables) evaluated associations between headphone-use patterns and outcomes. A *P*-value < 0.05 was considered statistically significant.

## Results

A total of 250 students participated in the study, comprising 121 (48.4%) Medical (MBBS), 56 (22.4%) Pharmacy, and 73 (29.2%) Nursing students. The mean age was 20.8 ± 2.1 years, with a range of 18-30 years. Table 1 presents the demographic profile across academic programs. Participants were primarily enrolled in Medical (MBBS), Pharmacy, and Nursing programs, with Medical (MBBS) students constituting the largest subgroup. Gender distribution differed significantly among courses (χ² = 92.2, *P* < 0.001).

**Table 1:** Distribution of students by program, gender, and age (n = 250). The chi-square test was used to assess differences in gender distribution across academic programs.

Academic program	Number of students (*n*)	Percentage (%)	Gender distribution (M/F)	Average age (Years)	Test statistic (χ²)	*P*-value
Medical	121	48.4	74/47	20.9 ± 1.8	χ² = 92.2	<0.001
Pharmacy	56	22.4	42/14	20.2 ± 2.3		
Nursing	73	29.2	0/73	21.5 ± 1.6		

**Figure 1:**
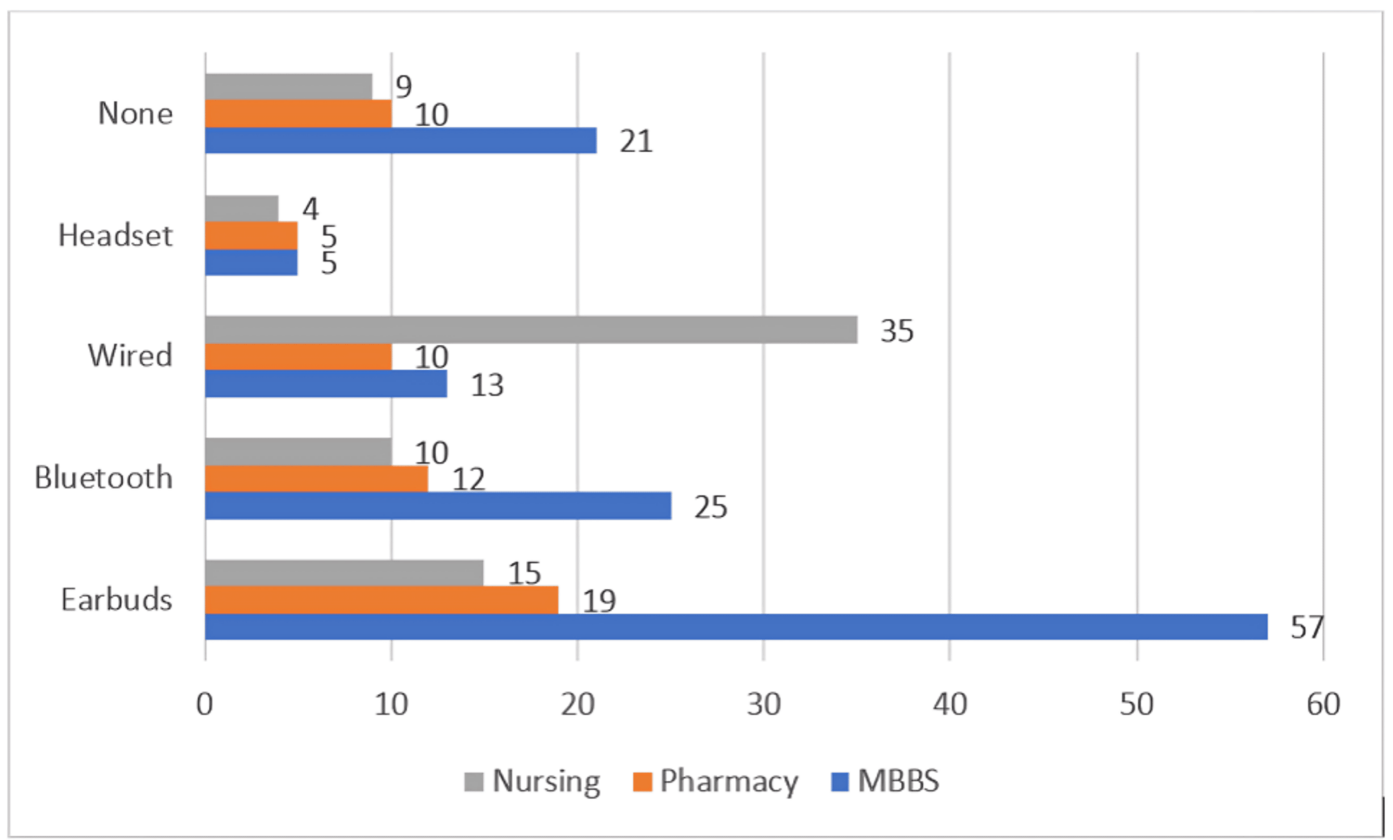
Distribution of headphone/earphone types by academic program (χ² = 40.88, P < 0.001).

Out of the 250 participants, 210 students (84%) reported regular use of headphones. Among these 210 students, earbuds were the most common device (91, 43.3%), followed by wired earphones (58, 27.6%), Bluetooth earphones (47, 22.4%) and headsets (14, 6.7%). The distribution of device preferences varied across academic programs (Table 2, Figure 1). Medical (MBBS) students predominantly used earbuds (57, 47.1%), whereas nursing students showed a marked preference for wired earphones (35, 47.9%). Pharmacy students demonstrated a more balanced distribution across device types, though earbuds remained the most popular (19, 33.9%).

**Table 2:** Distribution of device types used among healthcare students by course (n, %). The chi-square test was used to assess the association between academic program and device type.

Course	Earbuds, *n* (%)	Bluetooth, *n* (%)	Wired, *n* (%)	Headset, *n* (%)	None, *n* (%)	Test statistic (χ²)	*P*-value
Medical	57 (47.1)	25 (20.7)	13 (10.7)	5 (4.1)	21 (17.4)	χ² = 40.88	<0.001
Pharmacy	19 (33.9)	12 (21.4)	10 (17.9)	5 (8.9)	10 (17.9)		
Nursing	15 (20.5)	10 (13.7)	35 (47.9)	4 (5.5)	9 (12.3)		

A chi-square test of independence demonstrated a statistically significant association between academic program and headphone type (χ² = 40.88, df = 8, *P* < 0.001), indicating that the choice of earphones/headphones differed significantly across Medical (MBBS), Pharmacy, and Nursing students.

**Figure 2:**
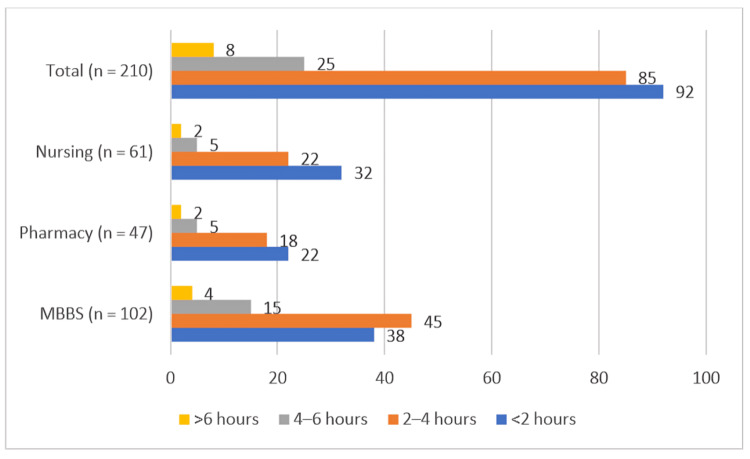
Distribution of daily headphone usage durations across academic programs. Statistical comparison using the chi-square test showed no significant difference (χ² = 4.33, df = 6, *P* = 0.632).

Daily headphone usage among regular users (*n* = 210) varied considerably (Figure 2). The majority listened for less than four hours per day - 92 (43.8%) used headphones for <2 hours, while 85 (40.5%) used them for 2-4 hours. A smaller proportion reported 4-6 hours (25, 11.9%) and >6 hours (8, 3.8%).

When compared across academic programs, the distribution of listening duration did not differ significantly (χ² = 4.33, df = 6, *P* = 0.632) (Table 3).

**Table 3:** Distribution of daily headphone usage across academic programs (n = 210). The chi-square test of independence was used to compare headphone usage duration across academic programs; the difference was not statistically significant.

Program	<2 hours, *n* (%)	2-4 hours, *n* (%)	4–6 hours, *n* (%)	>6 hours, *n* (%)	Total	Test statistic (χ²)	*P*-value
Medical	38 (37.3)	45 (44.1)	15 (14.7)	4 (3.9)	102	χ² = 4.33	0.632
Pharmacy	22 (46.8)	18 (38.3)	5 (10.6)	2 (4.3)	47	-	-
Nursing	32 (52.5)	22 (36.1)	5 (8.2)	2 (3.3)	61	-	-
Total	92 (43.8)	85 (40.5)	25 (11.9)	8 (3.8)	210	-	-

**Figure 3:**
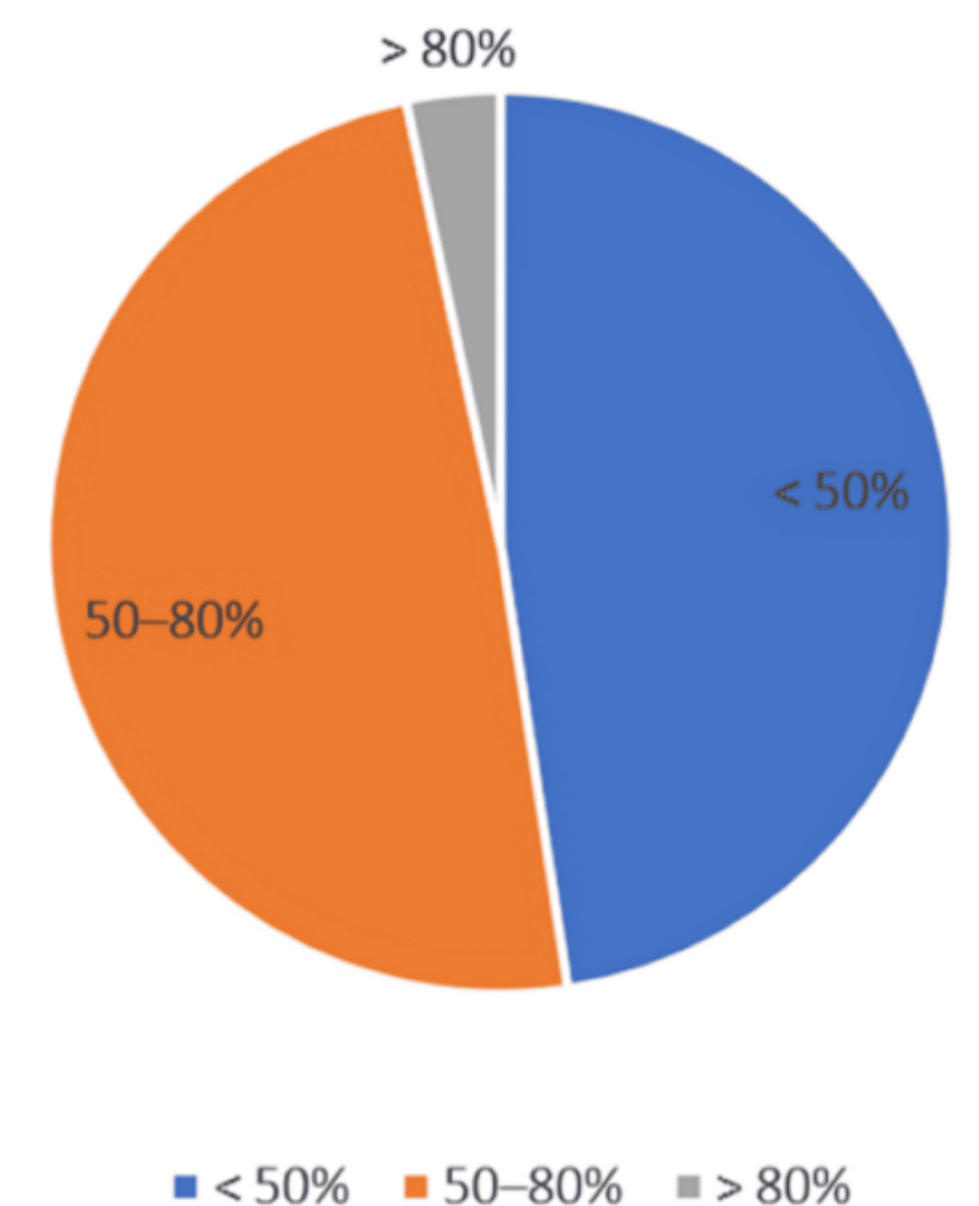
Preferred listening volume levels among students (n = 210).

In terms of preferred listening volume, 100 (47.6%) participants reported using their devices at less than 50% of the maximum volume, 103 (49.0%) listened at moderate levels between 50%-80%, and 7 (3.4%) reported listening at high volumes exceeding 80% (Figure 3).

**Figure 4:**
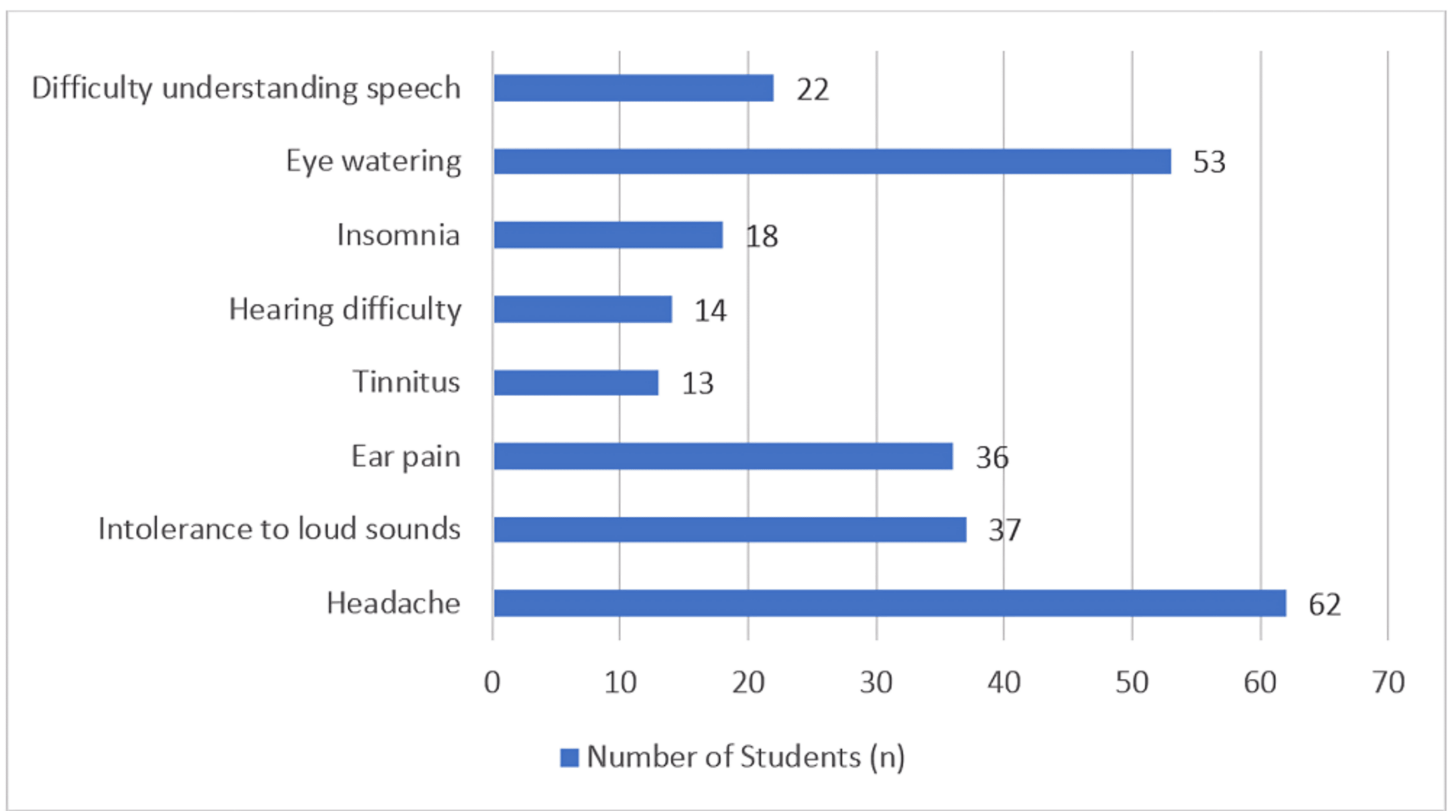
Frequency of hearing-related and associated symptoms among students (n = 250). Values are descriptive; no statistical test applied.

Several hearing-related and associated symptoms were reported (Figure 4). The most frequently reported complaint was headache (62, 24.8%), followed by eye watering (53, 21.2%). Other common symptoms included intolerance to loud sounds (37, 14.8%) and ear pain (36, 14.4%). Less frequently reported complaints were difficulty understanding speech (22, 8.8%), insomnia (18, 7.2%), hearing difficulty (14, 5.6%), and tinnitus (13, 5.2%).

**Figure 5:**
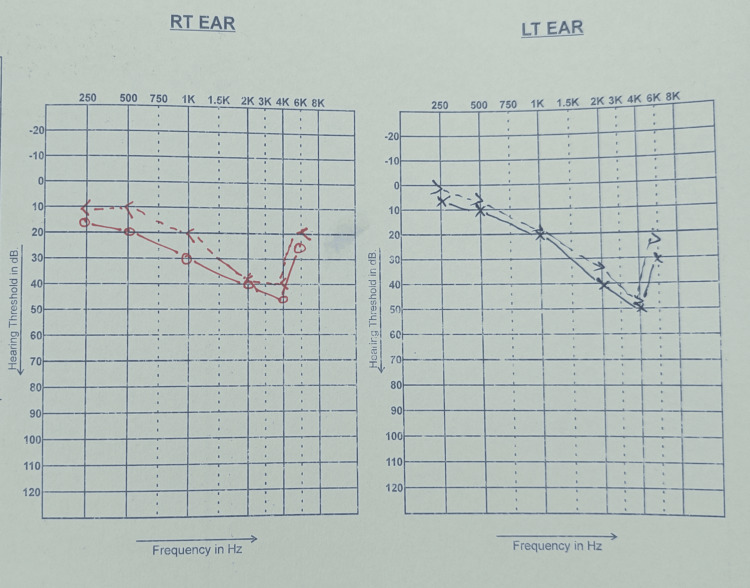
Audiogram demonstrating a 4-6 kHz notching pattern typical of noise-induced hearing loss.

Among the 250 participants who underwent PTA, SNHL was identified in 9 cases, corresponding to a prevalence of 3.6% (95% CI: 1.9%-6.7%). Regarding severity, 7 participants (77.8%) demonstrated mild SNHL, while 2 participants (22.2%) exhibited moderate impairment. No cases of severe or profound SNHL were detected. The mean hearing threshold at 4 kHz in SNHL cases was 32.5 ± 6.8 dB HL, suggesting early high-frequency involvement (Figure 5). Of these, 7 (77.8%) reported at least one symptom, while 2 (22.2%) were asymptomatic. In comparison, among the 241 students without SNHL, 146 (60.6%) reported symptoms and 95 (39.4%) did not. Although symptoms were more frequently reported in the SNHL group (77.8% vs. 60.6%), the difference was not statistically significant (Fisher’s exact test, *P* = 0.28) (Table 4).

**Table 4:** Association between hearing loss and self-reported symptoms (n = 250). Fisher’s exact test was used due to small cell counts. Although symptoms were more frequent among students with SNHL (77.8% vs. 60.6%), the association was not statistically significant (*P* = 0.28). SNHL, sensorineural hearing loss

Hearing status (PTA)	≥1 symptom, *n* (%)	No symptom, *n* (%)	Total	*P*-value
SNHL present	7 (77.8)	2 (22.2)	9	0.28
SNHL absent	146 (60.6)	95 (39.4)	241	-
Total	153 (61.2)	97 (38.8)	250	-

Symptom-specific analyses using Fisher’s exact test revealed no statistically significant associations between hearing loss and individual symptoms (all *P* > 0.05). However, a non-significant trend was observed for tinnitus, which was reported by 2 of 9 students with hearing loss (22.2%) compared to 11 of 241 students without hearing loss (4.6%) (*P* = 0.067).

## Discussion

Hearing loss is a significant public health issue that can profoundly impact quality of life, affecting communication, education, employment opportunities, and social interactions [9]. Our study included 250 students aged 18 to 30 years (mean 20.8 ± 2.1 years), which aligns closely with comparable studies [4,10-12]. A total of 210 (84%) participants reported regular headphone use, corroborating the findings of Rekha et al., who reported 86.1% prevalence of personal listening device usage among medical students [13]. Most participants in our study preferred insert-type listening devices, a particularly concerning trend since intra-canal placement delivers sound directly to the tympanic membrane, potentially causing greater harm than over-ear alternatives. Smartphones were the most widely used source. Multiple studies have established that prolonged earphone use at hazardous noise levels significantly increases the risk of hearing damage [14,15].

Our findings highlight a concerning pattern in listening behaviors. Nearly half of the students, 103 (49.0%), listened at moderate volumes (50%-80% of maximum), while a smaller but important subset (7, 3.4%) exceeded 80% of maximum output. Previous studies suggest that exposure at these levels, especially over extended durations, significantly increases the risk of noise-induced hearing loss (NIHL) [3,15]. We observed a clear dose-response relationship: students who listened at higher volumes and for longer periods reported more tinnitus, hearing difficulty, and intolerance to loud sounds, echoing evidence that unsafe listening practices can cause early auditory damage even in young adults. Symptom prevalence rose dramatically when high listening volumes were combined with prolonged use: every student who listened at >80% volume for more than four hours per day reported multiple symptoms, whereas those who listened for the same duration but kept volumes below 50% experienced far fewer complaints. This underscores the critical role of volume regulation, even in the context of longer exposure. Duration itself, however, was also a key determinant of risk. Students who listened for more than two hours daily were about three times more likely to report symptoms than those with shorter exposure, and this effect was most pronounced among Medical (MBBS) students - nearly one-fifth listened for more than four hours per day, reflecting the demands of their coursework. In contrast, nursing students tended to limit their use to under two hours at safer volume levels. Taken together, these findings suggest that while both exposure time and intensity contribute to auditory risk, controlling volume may be the more actionable strategy for preventing headphone-related hearing problems.

Importantly, these self-reported listening behaviors showed a significant correlation with audiometric findings. PTA identified SNHL in 9 participants (3.6%). Most cases were mild, with only two showing moderate loss. The clinical implications of such losses are considerable: mild SNHL (20-40 dB HL) results in up to 50% speech perception loss, moderate SNHL (41-60 dB HL) leads to marked distortion of conversation, and severe SNHL (>60 dB HL) renders speech largely inaudible [16]. Although the prevalence in our study was relatively low, it remains noteworthy when compared to prior studies - Manisha et al. reported a 36.1% prevalence of hearing loss among students [12], while a recent meta-analysis by Kornisch et al. identified a pooled prevalence of 19% in college populations [8], suggesting variability across different contexts and populations.

Our audiometric findings also provide objective evidence of noise-induced hearing damage. In particular, 8 of 9 SNHL cases (72.7%) demonstrated the characteristic notching pattern at 4-6 kHz, which is pathognomonic of NIHL. The disproportionate representation of Medical (MBBS) students (55.6% of SNHL cases) further reinforces the association between academic demands, prolonged headphone use, and recreational listening habits. Similar patterns of higher susceptibility among medical students have recently been reported by [17], supporting the view that this subgroup may be at uniquely elevated risk.

Several limitations should be considered. Exposure data were self-reported, introducing potential recall bias and misclassification of listening habits. The cross-sectional design precludes causal inferences about the progression of hearing damage. Finally, the study was conducted at a single institution and may not generalize to the broader student population. Future multicenter, longitudinal studies incorporating objective exposure monitoring will help clarify the temporal relationship between unsafe listening practices and auditory decline.

## Conclusions

This study highlights unsafe headphone use as an emerging risk factor for early auditory symptoms and measurable hearing loss in young adults. Prolonged high-volume listening was consistently linked with tinnitus, hearing difficulty, and intolerance to loud sounds, while audiometry revealed mild sensorineural losses with characteristic noise-induced notching in a subset of students. Medical students appeared particularly vulnerable, reflecting greater exposure and academic demands. These findings emphasize the importance of preventive strategies such as education on safe listening practices, awareness campaigns, routine auditory screening for high-risk groups, and promotion of volume-limiting measures. Future multicenter longitudinal studies with larger samples are warranted to better define the long-term risks of personal listening device use and guide evidence-based policy interventions.

## Appendices

Appendix 

**Table 5:** Performa (Survey Tool / Data Collection Sheet) Survey questionnaire developed by Dr. P. Chakraborty and Dr. Tamanna Pravin.

Q. No.	Question	Options (tick ✓ one)
1	Age: _______ Sex: ☐ Male ☐ Female	
2	Course & Year: ___________________	
3	Daily mobile/iPad screen time	☐ < 4 h ☐ 4–6 h ☐ 6–8 h ☐ > 8 h
4	Use of Headphones/Earbuds/Bluetooth	☐ Yes ☐ No
5	Daily headphone use duration	☐ < 2 h ☐ 2–4 h ☐ 4–6 h ☐ > 6 h
6	Usual volume level	☐ < 50% ☐ 50–80% ☐ > 80%
7	Do you play mobile games?	☐ Yes ☐ No
7a	If Yes, gaming duration	☐ < 4 h ☐ 4–6 h ☐ 6–8 h ☐ > 8 h
8	Game played most often	_________________________
9	Symptoms experienced (tick all that apply)	☐ Tinnitus ☐ Hearing difficulty ☐ Loud sound intolerance ☐ Headache ☐ Ear pain ☐ Eye watering ☐ Insomnia
10	Any ear problem (past or present)	☐ Yes (specify) __________ ☐ No
11	History of ear surgery	☐ Yes ☐ No
12	Usual bedtime	___________
13	Sleep duration in 24 h	_______ hours
14	Willing for free hearing assessment (PTA / Impedance audiometry)?	☐ Yes ☐ No
